# Identification of Hub Genes Associated With Sensitivity of 5-Fluorouracil Based Chemotherapy for Colorectal Cancer by Integrated Bioinformatics Analysis

**DOI:** 10.3389/fonc.2021.604315

**Published:** 2021-04-12

**Authors:** Ya Wang, Qunhui Wei, Yuqiao Chen, Shichao Long, Yuanbing Yao, Kai Fu

**Affiliations:** ^1^ Institute of Molecular Precision Medicine and Hunan Key Laboratory of Molecular Precision Medicine, Department of General Surgery, Xiangya Hospital, Central South University, Changsha, China; ^2^ Center for Medical Genetics & Hunan Key Laboratory of Medical Genetics, School of Life Sciences, Central South University, Changsha, China; ^3^ Hunan Key Laboratory of Animal Models for Human Diseases, Central South University, Changsha, China; ^4^ National Clinical Research Center for Geriatric Disorders, Changsha, China; ^5^ Hunan Key Laboratory of Aging Biology, Xiangya Hospital, Central South University, Changsha, China

**Keywords:** colorectal cancer, 5-fluorouracil, chemotherapy sensitive, weighted gene co-expression network analysis, biomarker

## Abstract

Colorectal cancer (CRC) is one of the most common malignant tumors. 5-fluorouracil (5-FU) has been used for the standard first-line treatment for CRC patients for several decades. Although 5-FU based chemotherapy has increased overall survival (OS) of CRC patients, the resistance of CRC to 5-FU based chemotherapy is the principal cause for treatment failure. Thus, identifying novel biomarkers to predict response to 5-FU based chemotherapy is urgently needed. In the present study, the gene expression profile of GSE3964 from the Gene Expression Omnibus database was used to explore the potential genes related to intrinsic resistance to 5-FU. A gene module containing 81 genes was found to have the highest correlation with chemotherapy response using Weighted Gene Co-expression Network Analysis (WGCNA). Then a protein-protein interaction (PPI) network was constructed and ten hub genes (*TGFBI*, *NID*, *LEPREL2*, *COL11A1*, *CYR61*, *PCOLCE*, *IGFBP7*, *COL4A2*, *CSPG2*, and *VTN*) were identified using the CytoHubba plugin of Cytoscape. Seven of these hub genes showed significant differences in expression between chemotherapy-sensitive and chemotherapy-resistant samples. The prognostic value of these seven genes was evaluated using TCGA COAD (Colorectal Adenocarcinoma) data. The results showed that TGFBI was highly expressed in chemotherapy-sensitive patients, and patients with high TGFBI expression have better survival.

## Introduction

CRC is one of the most common malignant tumors and the second cause of tumor-related mortality worldwide (1, 2). By 2030, the global burden of CRC is expected to increase by 60%, with 2.2 million new cases and 1.1 million deaths (3). The 5-year survival for CRC patients with local tumor is 90.3%, and 70.4% for patients with locally advanced disease, which declines to 12.5% for patients with metastatic disease (4). Surgery is highly recommended for early CRC and locally advanced CRC (5, 6). However, half of the patients treated with surgery will suffer a recurrence within 3 years after surgery (7). For patients with stage III and some stage II CRC, chemotherapy followed by surgery is given for about six months to reduce the risk of recurrence (8).

Over the last few decades, substantial progress has been made in the development of new treatment regimens that fundamentally increase the overall survival (OS) of CRC patients. Patients with stage III or high-risk stage II CRC benefit from the use of adjuvant chemotherapy with5-FU-based regimens (9, 10). Although most patients can benefit from chemotherapy, others may suffer ineffective chemotherapy for several cycles until the treatment effects are determined, which usually leads to adverse, life-threatening side effects (11, 12). The resistance of CRC to 5-FU based chemotherapy is the principal cause for treatment failure. Thus, the stratification of chemotherapy response based on biological characteristics is critical for individualized treatment. Identifying novel biomarkers to predict response to 5-FU based chemotherapy is urgently needed.

Human tumors become resistant to treatment in the presence of a drug, that is, tumors possessing innate resistance to drugs. Innate resistance is usually detected in the early stages of drug development or early clinical trials of biological effects. However, sometimes innate resistance can’t be found until retrospective analysis of *in vivo* studies (13).

Some biomarkers that predict the response of 5-FU based therapy for CRC patients have been identified. Low expression of thymidylate synthase (TS), an enzyme encoded by *TYMS* gene, was associated with increased sensitivity to 5-FU based therapy (14, 15). Several studies have indicated that the expression of dihydropyridine dehydrogenase (DPD) which is encoded by *DPYD* gene is a predictive marker for both the effectiveness and toxicity of 5-FU treatment (16). High DPD activity in tumor tissue might be associated with the drug resistance by reducing the cytotoxic effects of 5 FU (16). In addition, DPD level affects 5-FU catabolism, low DPD level leading to an effective accumulation of the drug inside cell through reducing 5-FU catabolism (17). It has been reported the range of *DPYD* expression in CRC tissues which were nonresponsive to 5-FU was much broader than that of the responding CRC tissues (18). Thymidine phosphorylase (TP), encoded by *TYMP* gene, has been found to be a useful marker for predicting the effectiveness of 5-FU based chemotherapy (19). There is a correlation between low TP expression and improved treatment outcomes, low TP expression predicting a good response to 5-FU chemotherapy (20, 21). However, some other studies indicated the opposite conclusion. The cells with higher TP expression may be related to increased sensitivity to 5-FU (16). Besides, membrane transporter proteins are involved in chemoresistance mechanisms by transporting drugs out of the cell, thereby resulting in chemotherapy failure. ATP-binding cassette (ABC) transporters belong to membrane transporter proteins. Several ABC transporters related to 5-FU resistance of CRC patients have been identified, such as ABCB5 (22), ABCC11 (23). Although some proteins and mechanisms associated with 5-FU resistance have been reported, more biomarkers and related mechanisms of 5-FU resistance remain to be further studied. In the present study, we aimed to explore novel biomarkers for predicting intrinsic resistance of CRC patients to 5-FU.

## Materials and Methods

### Data Collection

**Figure 1:**
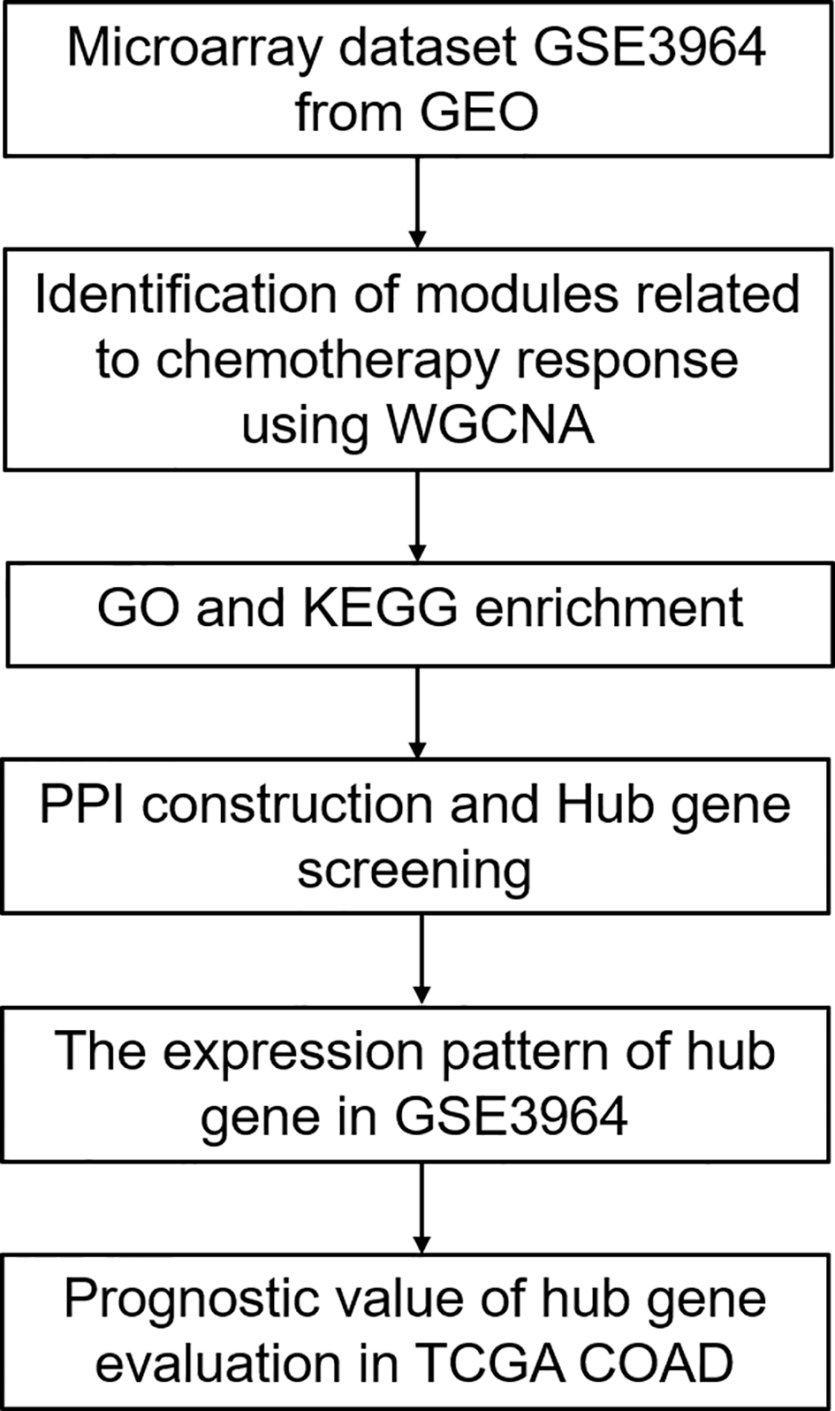
The workflow for analyzing the gene related to chemotherapy response in CRC.

A flowchart of this study is presented in **Figure 1**. Gene expression profiles of Dataset GSE3964 were downloaded from Gene Expression Omnibus (GEO) database (https://www.ncbi.nlm.nih.gov/geo/query/acc.cgi?acc=GSE3964). This dataset contains expression profiling of clinical samples collected from CRC patients before the exposure to 5-FU based combined chemotherapy. Analysis of gene expression profiles between chemotherapy-sensitive patients and chemotherapy-resistant patients may identify biomarkers associated with innate tumor drug responses. Another gene expression profiles of CRC patients undergoing chemotherapy and corresponding clinical information were downloaded from TCGA COAD (https://portal.gdc.cancer.gov/). Gene expression profiles of Dataset GSE19860 without z-score normalized (https://www.ncbi.nlm.nih.gov/geo/query/acc.cgi?acc=GSE19860) was used to verify the expression patterns of the screened hub genes. This dataset contains responders and non-responders who received modified FOLFOX6 therapy.

### Weighted Gene Co-Expression Network Analysis (WGCNA)

The gene expression profile of GSE3964 was constructed to gene co-expression networks using the WGCNA package in R to explore the modules of highly correlated genes among samples for relating modules to external sample traits (24). A weighted adjacency was constructed through calculating Pearson correlations of all gene pairs. Soft power β= 4 was selected to construct a standard scale-free network. The similarity matrix which is done by Pearson correlation of all gene pairs was transformed into a topological overlap matrix (TOM) as well as the corresponding dissimilarity (1- TOM). Then a hierarchical clustering dendrogram of the 1-TOM matrix was used to classify the similar gene expression into different gene co-expression modules. Afterward, the module-clinical trait association was calculated to identify functional modules in a co-expression network. The module with a high correlation coefficient was regarded to be associated with clinical traits and was selected for further analysis.

### GO and KEGG Functional Enrichment Analyses

Gene ontology (GO) and Kyoto Encyclopedia of Genes and Genomes (KEGG) enrichment analyses for genes in functional modules were performed using an R package “clusterProfiler”. GO annotation is based on three categories, including biological processes (BP), cellular compartments (CC), and molecular functions (MF). Terms in GO and KEGG with a false discovery rate (FDR) < 0.05 were considered significantly enriched and were visualized by R package “ggplot2”.

### Protein-Protein Interaction Network Construction and Hub Gene Screening

The protein-protein interaction (PPI) network of genes in functional modules was constructed with an online STRING database (https://string-db.org), and an interaction with a combined score > 0.4 was considered as statistical significance. Cytoscape, an open-source bioinformatics software platform, was used to visualize molecular interaction networks. Hub genes were identified using the DMNC algorithm of cytoHubba plugin in Cytoscape.

### Verification of the Expression Patters and Prognostic Values of Hub Genes

The expression of the top ten hub genes between chemotherapy-sensitive and chemotherapy-resistant samples was analyzed. Gene with *p* < 0.05 is considered a significantly differentially expressed gene between the chemotherapy-resistant and chemotherapy-sensitive group. Then prognostic values of significantly differentially expressed hub genes were evaluated in CRC patients undergoing chemotherapy from TCGA COAD. Kaplan-Meier survival analysis was performed using the “survival” R package based on the median value of each gene. *p* < 0.05 is considered a statistically significance.

### Cell Culture and Transfection

HCT116 and DLD1 cells (purchased from ATCC) were cultured in DMEM medium (Cellmax) containing 10% fetal calf serum (BI), and 100 U/ml each of penicillin and strepcomycin (BI) at 37°C with 5% CO_2_. Cells were transfected with si-RNAs or expression plasmids using Lipofectamine 2000 reagent (Invitrogen, USA) according to the manufacturer’s instructions.

### Establishment of 5-FU Resistant Cells

To establish 5-FU resistant cells, cells were treated with a high concentration of 5-FU for 24h, then the media was replaced with fresh media containing a low concentration of 5-FU. After 2 weeks of treatment at the low concentration, increase 1.5 times of the dose, and repeat the same. 5-FU resistant HCT116 cells were generated by treating its parental cells with 40µM 5-FU for 24h, then treating the cells with 0.3125 µM 5-FU and increasing 1.5 times of the dose. 5-FU resistant DLD1 cells were generated by treating its parental cells with 100µM 5-FU for 24h, then treating the cells with 1.25µM 5-FU and increasing 1.5 times of the dose. The 5-FU resistant HCT116 cells and 5-FU resistant DLD1 cells were obtained by continuous exposure to gradually increased concentrations of 5-FU for four months.

### MTT Assay

HCT116 or DLD1 cells were washed with DMEM without phenol red and incubated with MTT (3-(4,5)-dimethylthiahiazo(-z-y1)-3,5-di-phenytetrazoliumromide) at the concentration of 0.5 mg/ml in DMEM without phenol red. Four hours after incubation, the media were dumped off and the formazan crystals were dissolved in dimethyl sulfoxide (DMSO). The optical density (OD) was measured by a photometer at 490 nm. The data were normalized to control and the ratios were presented as mean ± SE with three experiments.

### Immunoblot Analysis

Proteins were separated by 9% SDS-PAGE and then transferred onto a nitrocellulose membrane (pall). The following primary antibodies were used: anti-Beta Actin (Proteintech), anti-TGFBI (Proteintech). The following secondary antibody was used: Goat anti-mouse IgG-HRP antibody (Proteintech). The proteins were visualized using an ECL detection kit (GE).

### Plasmids

Full-length TGFBI DNA was amplified by PCR with the primer 5’-GATCTCGAGCTCAAGCTTCGAATTCCATGGCGCTCTTCGTGCGG-3’ and 5’-GATCTCGAGCTCAAGCTTCGAATTCCATGGCGCTCTTCGTGCGGTCAGTTATCTAGATCCGGTGGATCCCTAATGCTTCATCCTCTCTAATAACTTTTGATAGACAG-3’ using pOTB7-TGFBI (P14682, www.miaolingbio.com). pEGFP-C1 was linearized through EcoRI and BamHI. Then the TGFBI DNA was cloned into pEGFP-C1 through Gibson Assembly.

## Results

### Weighted Gene Co-Expression Network Analysis and Key Modules Identification

**Figure 2:**
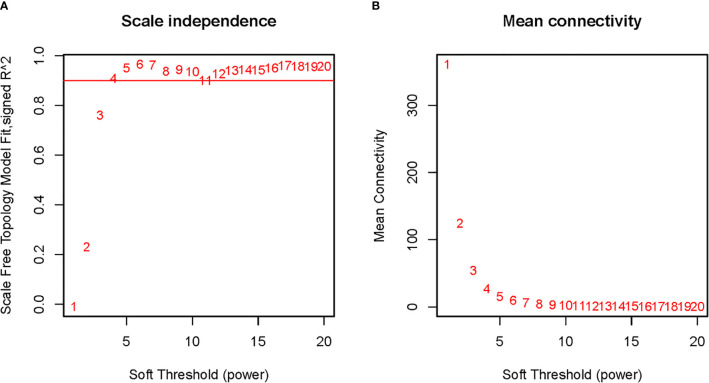
Determination of soft-thresholding power in weighted gene co-expression network analysis (WGCNA). **(A)** The scale-free fit index for various soft-thresholding powers; **(B)** The mean connectivity for various soft-thresholding powers.

**Figure 3:**
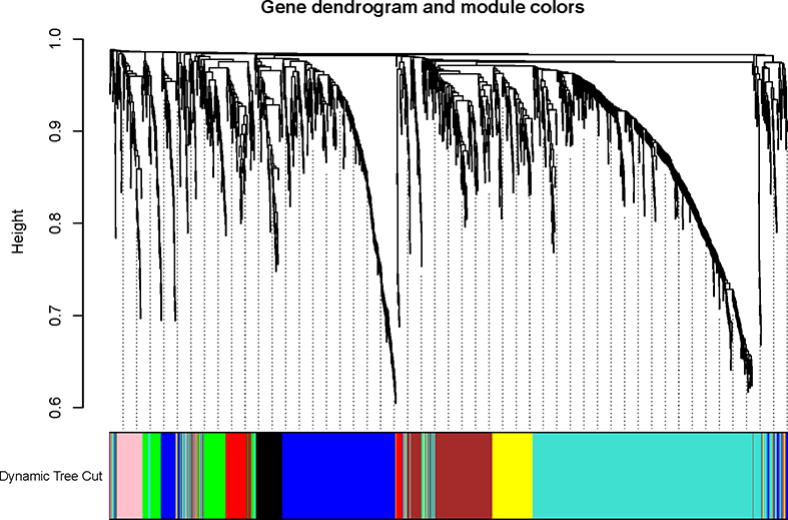
The Cluster dendrogram of co-expression network modules was ordered by a hierarchical clustering of genes based on the 1-TOM matrix. Each module was assigned to different colors.

**Figure 4:**
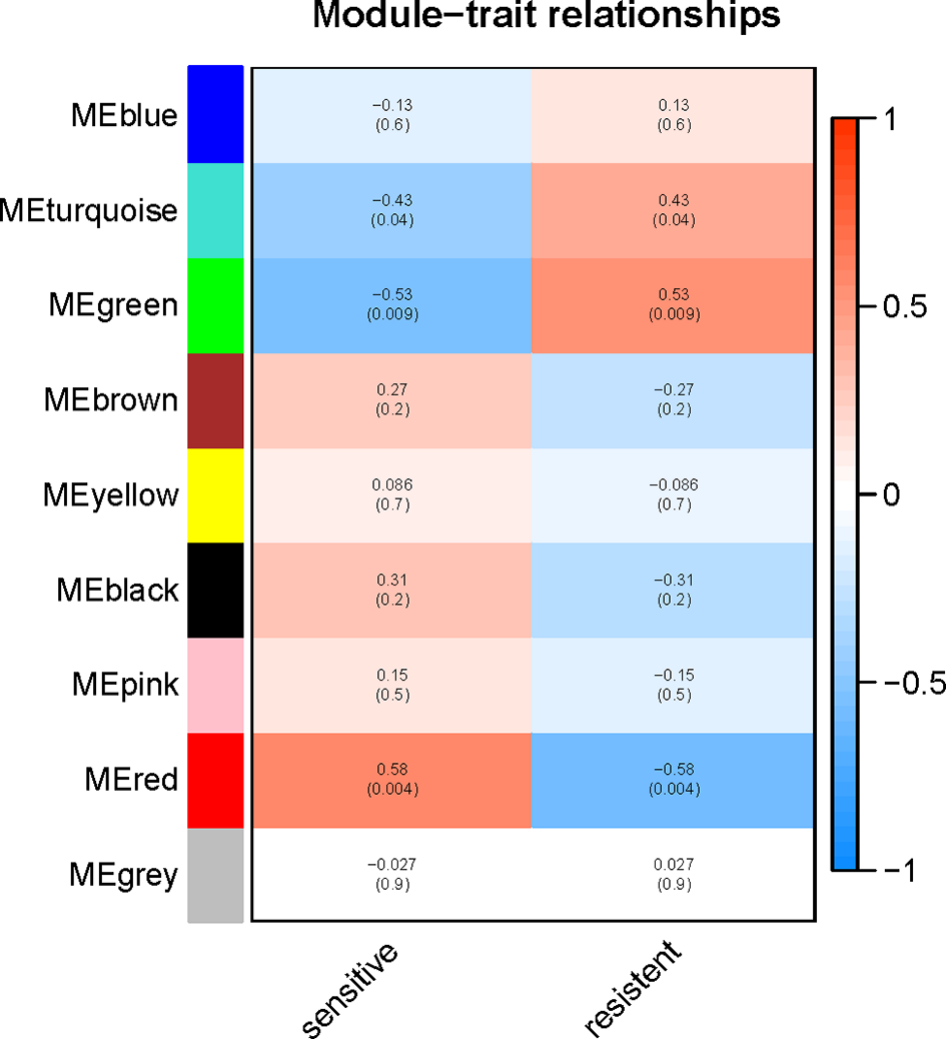
Relationships between the module and clinical traits. Each row represents a color module and column corresponds to a clinical trait (chemotherapy-sensitive or chemotherapy-resistant). Each cell contains the corresponding correlation and *p-*value.

**Figure 5:**
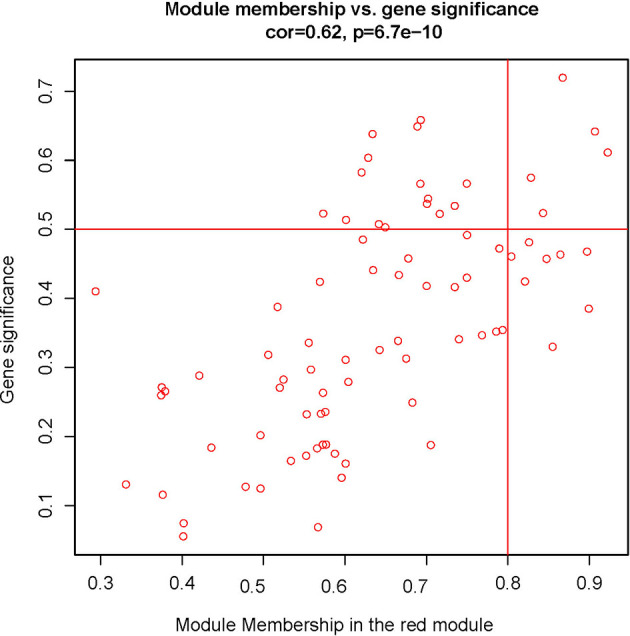
Scatter plots of the red module.

To explore the functional clusters related to chemotherapy response, the weighted gene co-expression network was constructed from GSE3964 datasets which containing 10 chemotherapy-sensitive and 13 chemotherapy-resistant samples. The included samples were clustered with the average linkage hierarchical clustering method. The power of β = 4 was selected as the soft-thresholding parameter to conduct a scale-free network (**Figure 2**). A total of 12 modules were identified with the average linkage hierarchical clustering (**Figure 3**). The size of each identified module was listed in **Supplementary Table 1**. To evaluate the association between each module and two clinical traits (chemotherapy-sensitive and chemotherapy-resistant), the heatmap of the module-trait relationship was plotted (**Figure 4**). The red module was found to have the highest correlation with chemotherapy response (**Figure 4**, r = 0.58, *p* = 0.004). The genes in the red module were highly correlated with the module (**Figure 5**, r = 0.62, *p* = 6.7×10^-10^).

### Functional and Pathway Enrichment Analysis

**Figure 6:**
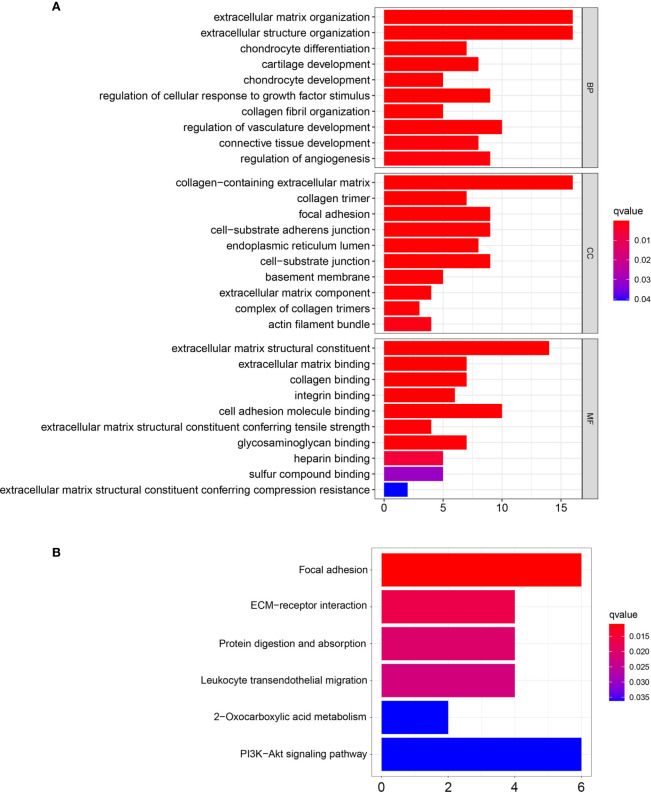
GO and KEGG enrichment analysis of the genes in the red module. **(A)** GO enrichment analysis based on biological processes (BP), cellular compartments (CC), and molecular functions (MF). **(B)** KEGG enrichment analysis.

To explore the potential function of the red module which had the highest correlation with chemotherapy response, GO and KEGG enrichment analysis was performed. For BP enrichment, the genes in the red module were mostly enriched in extracellular matrix organization and extracellular structure organization (**Figure 6A**). For CC enrichment, these genes were mainly involved in collagen-containing extracellular matrix and collagen trimer (**Figure 6A**). For MF enrichment, these genes were mainly enriched in extracellular matrix structural constituent and extracellular matrix binding (**Figure 6A**). For KEGG enrichment, these genes were mainly enriched in focal adhesion pathway and ECM-receptor interaction pathway (**Figure 6B**).

### PPI Network Construction and Hub Genes Screening

**Figure 7:**
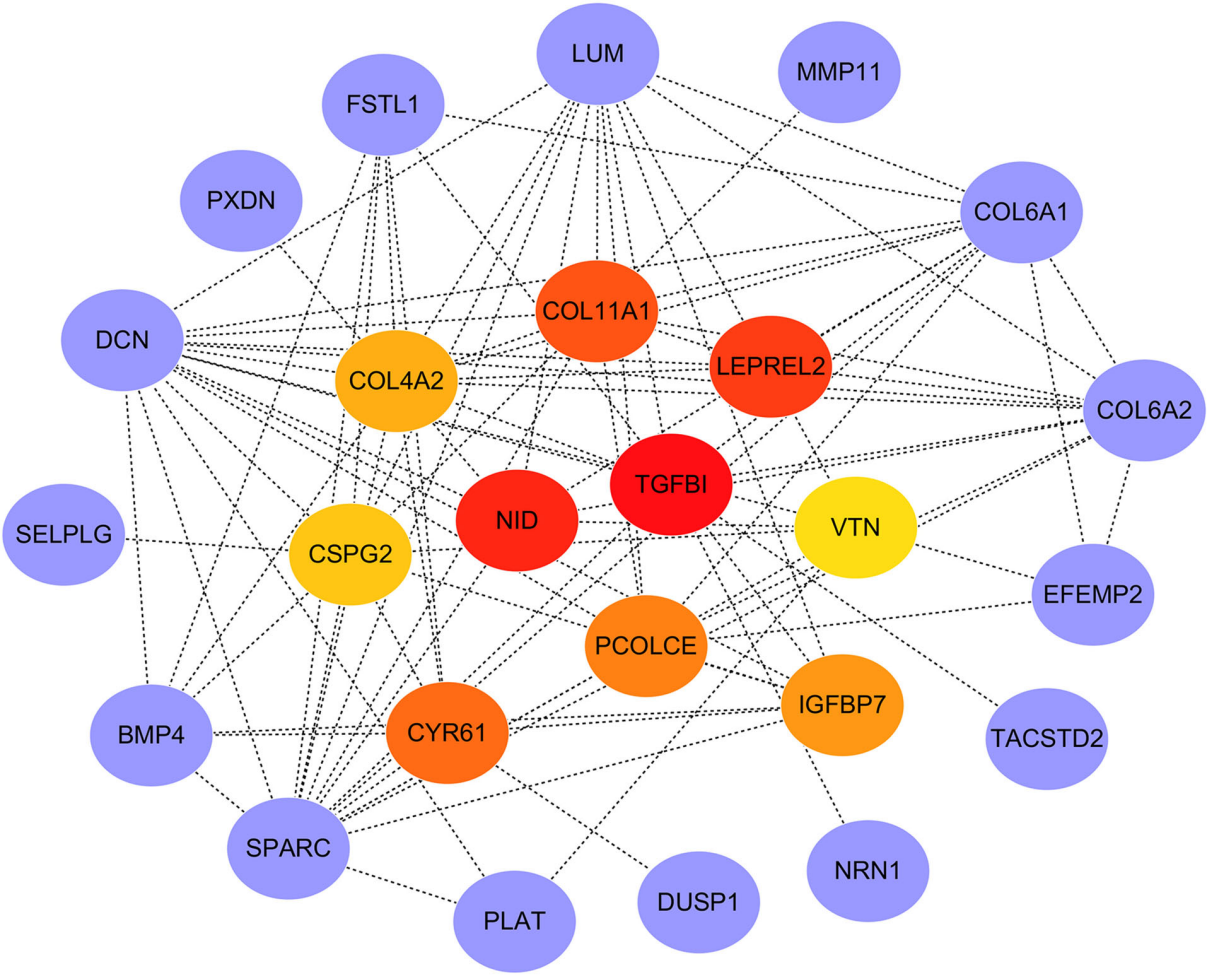
PPI network of genes in the red module and hub gene screening.

PPI network of the genes in the red module was constructed through the STRING database and visualized with Cytoscape software. The PPI network and hub genes identified from the network through the DMNC algorithm of CytoHubba plugin were shown in **Figure 7**. According to the DMNC scores, the top ten highest-scored genes, including *TGFBI, NID, LEPREL2, COL11A1, CYR61*, *PCOLCE*, *IGFBP7*, *COL4A2*, *CSPG2*, and *VTN*, were regarded as hub genes.

### Verification of the Expression Patterns and Prognostic Values of Hub Genes

**Figure 8:**
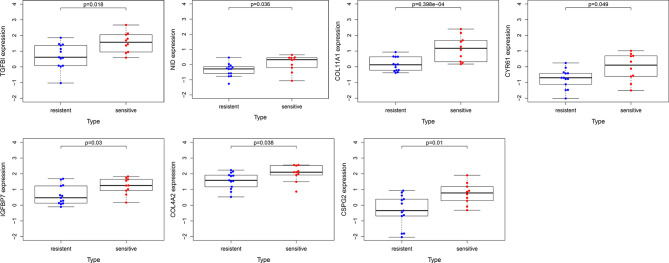
The expression of hub genes between chemotherapy-resistant samples and chemotherapy-sensitive samples.

**Figure 9:**
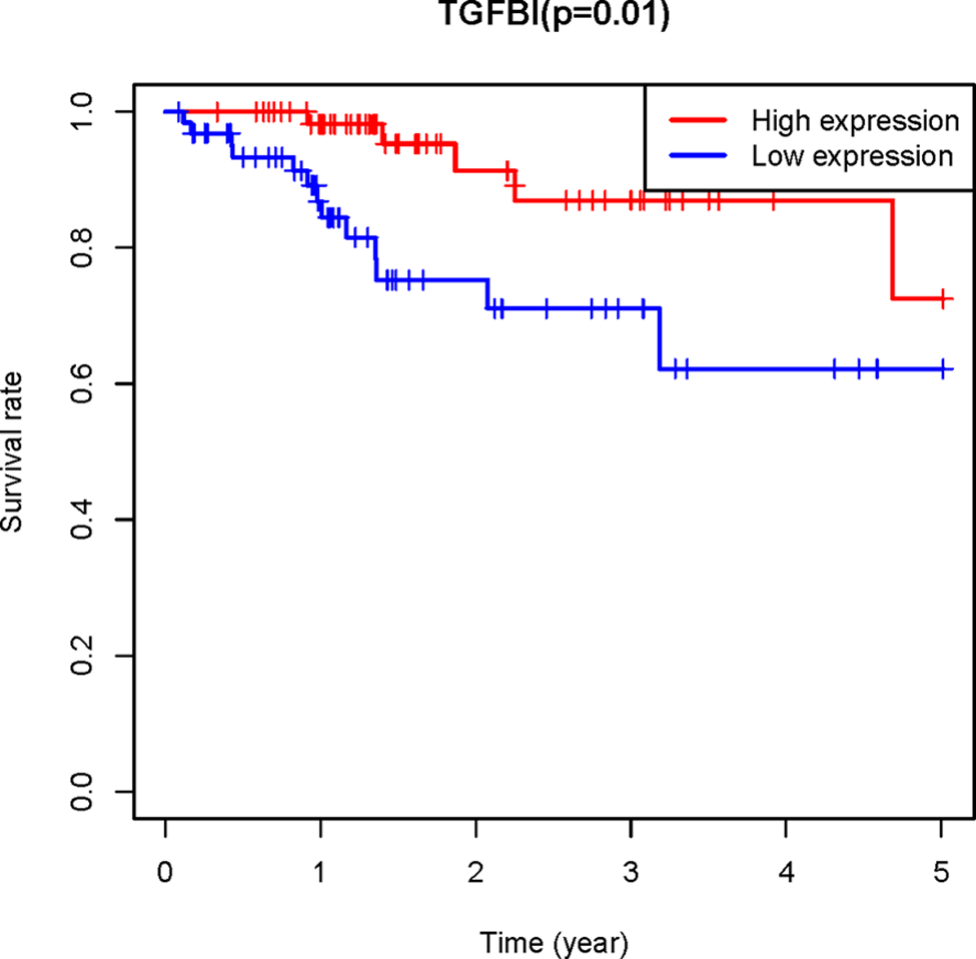
The Kaplan–Meier analysis. the Kaplan–Meier analysis of the patients from TCGA COAD receiving chemotherapy showed the survival of patients with high expression of TGFBI is better than those patients with low expression of TGFBI.

To confirm the reliability of the hub genes, the expression of the top ten hub genes between chemotherapy-sensitive and chemotherapy-resistant samples was plotted as a box plot graph. Seven hub genes showed significant differences between chemotherapy-sensitive and chemotherapy-resistant samples (**Figure 8**). To evaluate the prognostic value of the seven genes, the chemotherapy patients from TCGA COAD were stratified into a high-expression group and a low-expression group based on the median value of each gene. As shown in **Figure 9**, The survival of patients with high expression of TGFBI is better than those patients with low expression of TGFBI.

### Verification of the Relationship Between TGFBI level and 5-FU Sensitivity in Datasets and Cells

To further verify the relationship between TGFBI level and 5-FU sensitivity, samples from the GEO dataset (GSE19860) were analyzed. In line with our expectations, among the CRC patients who received modified FOLFOX6 therapy, responders also showed a higher level of TGFBI than non-responders (**Supplementary Figure 1**).

**Figure 10:**
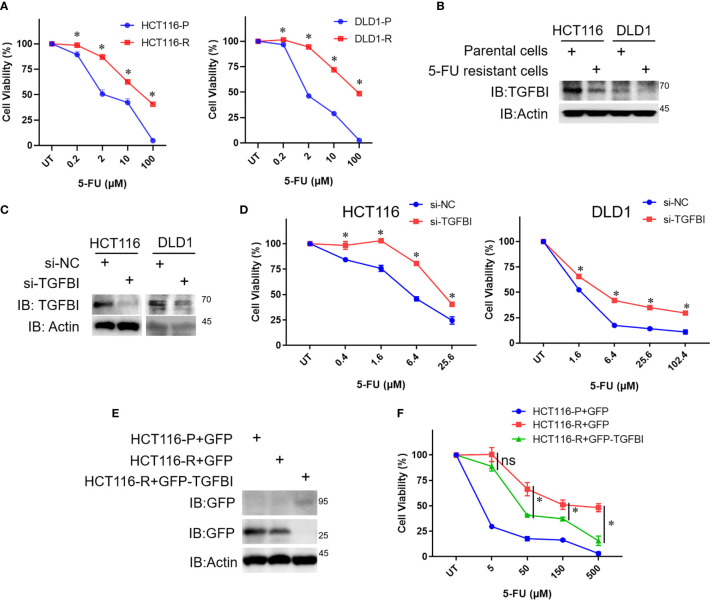
The relationship between TGFBI level and 5-FU sensitivity of CRC cells. **(A)** The parental cells and 5-FU resistant cells of HCT116 and DLD were treated with different concentrations of 5-FU for 72h, then the media were dumped off, cells were subjected to the 3-(4,5)-dimethylthiahiazo (-z-y1)-3,5-di-phenytetrazoliumromide (MTT) assay. Data were calculated from three independent experiments and analyzed by one-way analysis of variance (data shown are the means ± s.e.m., *p < 0.05). **(B)** Whole-cell lysates were derived from the parental cells and 5-FU resistant cells and immunoblotted (IB) for TGFBI, with actin as a loading control. **(C)** HCT116 cells or DLD1 cells were transfected with si-NC or si-TGFBI in day 0 and day 2, 8 h after the second transfection, some of the cells were harvested to detect TGFBI knockdown efficiency. The remaining cells were used for **(D)**. **(D)** Cells from **(C)**, which were transfected with si-NC or si-TGFBI twice, were treated with different concentrations of 5-FU for 72h, then cells were subjected to MTT assay. Data were calculated from three independent experiments and analyzed by one-way analysis of variance (data shown are the means ± s.e.m., *p < 0.05). **(E)** The parental cells of HCT116 were transfected with pEGFP, the resistant cells of HCT116 were transfected with pEGFP and pEGFP-TGFBI, respectively. Half of the cells were harvested to detect the expression of TGFBI using GFP-antibody after 24 h transfection. The remaining cells were used for **(F)**. **(F)** Cells from **(E)**, which were transfected with pEGFP or pEGFP-TGFBI were treated with different concentrations of 5-FU for 72h, then cells were subjected to MTT assay. Data were calculated from three independent experiments and analyzed by one-way analysis of variance (data shown are the means ± s.e.m., ns, no statistical significance by one-way analysis of variance, *p < 0.05).

To evaluate whether TGFBI mediates the response of CRC cells to 5-FU treatment. We generated the 5-FU resistant cells from the parental HCT116 cells and DLD1 cells, respectively by continuous exposure to gradually increased concentrations of 5-FU. Then the TGFBI levels were detected in the parental cells and 5-FU resistant cells. Cell viability of the parental cells and 5-FU resistant cells was detected using MTT assay. The IC_50_ of 5-FU was 4.032 µM, 40.085µM, 2.091µM, 70.820µM for the parental HCT116 cells, the 5-FU resistant HCT116 cells, the parental DLD1 cells, and the 5-FU resistant DLD1 cells (**Figure 10A**), which suggested that the resistant cells we obtained indeed more resistant to 5-FU. Consistent with our analysis, TGFBI levels are dramatically decreased in 5-FU resistant cell lines than that in the corresponding parental cells, both in HCT116 cells and DLD1 cells (**Figure 10B**). As shown in **Figures 10C, D**, knocking down TGFBI in HCT116 cells and DLD1 cells both led to decreased sensitivity to 5-FU treatment compared with control cells. To further verify our conclusion, a complementary experiment was carried out. GFP-TGFBI or GFP were transfected into 5-FU resistant HCT116 cells respectively, then the cell viability was detected after being treated with different concentrations of 5-FU. As we expected, compared with 5-FU resistant HCT116 cells overexpressing GFP, 5-FU resistant HCT116 cells overexpressing GFP-TGFBI showed increased sensitivity to 5-FU (**Figures 10E, F**).

## Discussion

5-FU has been used as the standard first-line treatment for CRC patients for several decades. To improve the anti-tumor activity of 5-FU and reduce drug resistance, some optimizing strategies have been adopted including 5-FU based combination therapy. Despite the encouraging progress in CRC treatment to data, failure of chemotherapy due to 5-FU resistance still occurs frequently. In the present study, the gene expression profile of CRC patients before their exposure to 5-FU based combined chemotherapy were analyzed to identify biomarkers related to intrinsic resistance to 5-FU based chemotherapy.

Potential gene modules related to response to 5-FU based chemotherapy were identified with WGCNA analysis. The red module was found to have the highest correlation with chemotherapy response. To further understand the potential function of genes among the red module, GO and KEGG enrichment analysis was performed. Functional and pathway enrichment analysis results showed the genes in the red module were mainly enriched in extracellular matrix organization and ECM-receptor interaction pathway.

PPI network of the genes in the red module was constructed and ten hub genes were screened through CytoHubba plugin in Cytoscape. Then the expression of the hub genes was confirmed in GSE3964. Among the ten hub genes, the expression of seven genes (*TGFBI*, *NID*, *COL11A1*, *CYR61*, *IGFBP7*, *COL4A2*, and *CSPG2*) showed significant differences between chemotherapy-sensitive and chemotherapy-resistant samples. To explore the prognostic value of these seven genes, Kaplan-Meier survival analysis was performed in CRC patients treated with 5-FU based chemotherapy from TCGA COAD. *TGFBI* was identified as a prognostic gene (*p* = 0.01), a high expression of which indicates a good prognosis (**Figure 8**). Also, the expression of TGFBI is higher in CRC patients who are sensitive to 5-FU based chemotherapy than those resistant to 5-FU based chemotherapy (**Figure 7**). These results suggested that TGFBI may act as a biomarker for predicting the response of 5-FU based chemotherapy for CRC patients.

TGFBI (transforming growth factor β-induced protein), encoded by *TGFBI* gene, was first identified in a human lung adenocarcinoma cell line A549 treated with TGFB (transforming growth factor β), it contains an RGD (Arg-Gly-Asp) motif that can serve as a ligand recognition site for integrins (25). TGFBI mediates cell adhesion to extracellular proteins including collagen, fibronectin, and laminins through integrin binding (26). Many reports have indicated that TGFBI functioned as a tumor suppressor. Down-regulation of TGFBI has been observed in various tumors. Immunochemistry results showed the expression of TGFBI in lung carcinomas was lower than normal tissues (27). TGFBI level got down-regulated due to promoter hypermethylation in ovarian carcinoma tissues (28). TGFBI promoter hypermethylation also occurs in lung and prostate cancer specimens (29). Overexpression of TGFBI in Chinese hamster ovary (CHO) cells resulted in a significant decrease in cell growth and tumor-forming ability of these cells in nude mice (30). TGFBI expression also reduced the metastatic potential of lung and breast tumor cells (31). TGFBI can facilitate TGFB-induced apoptosis through releasing RGD peptides when TGFBI normally undergoes carboxy-terminal processing (32). TGFBI deficiency predisposed mice to spontaneous tumor development (33). Besides, recovery of TGFBI expression in lung cancer cell H522 lacking endogenous TGFBI protein leads to a significant decrease in cell growth and a significantly higher sensitivity to apoptotic induction (27). These studies support that TGFBI functions as a tumor suppressor. However, controversy has arisen to the role of TGFBI in tumorigenesis. Multiple studies report a tumor-promoting function of TGFBI. TGFBI increased the metastatic potential of ovarian cancer cells, and TGFBI may be a potential therapeutic target against ovarian cancer (34). It has been suggested that TGFBI plays a dual role in ovarian cancer and can act both as a tumor suppressor or tumor promoter depending on the tumor microenvironment (35). A study suggested TGFBI may play a pro-tumor or anti-tumor role, depending on the integrins to which it binds on the cell surface (36).

In addition to the dual role in tumor progression, the expression of TGFBI has also been associated with chemotherapeutic drug sensitivity. Loss of TGFBI induced specific resistance to paclitaxel in ovarian cancer cells, and paclitaxel-resistant cells treated with recombinant TGFBI protein show restoration of paclitaxel sensitivity (37). Immunohistochemistry results in non-small cell lung cancer (NSCLC) clinical samples suggested there was a strong association between elevated TGFBI expression and the response to chemotherapy (38). Human NSCLC cells overexpressing TGFBI displayed increased sensitivity to etoposide, paclitaxel, cisplatin, and gemcitabine (38). High TGFBI level was associated with longer survival in lung squamous cell carcinomas patients received adjuvant platinum-based chemotherapy (39). The overexpression of TGFBI sensitized the nasopharyngeal carcinoma (NPC) cells to cisplatin (40). It has been reported that the TGF-β pathway is activated by 5-FU treatment in drug-resistant colorectal carcinoma cells (41). In the present study, we associated the expression level of TGFBI with the sensitivity of 5-FU based chemotherapy for CRC for the first time. Our results suggested that CRC patients with high expression of TFGBI indicated increased sensitivity of 5-FU based chemotherapy and improved survival. The conclusion was further confirmed in an independent dataset (GSE19860). Furthermore, experiments *in vitro* also support the conclusion. TGFBI levels were dramatically decreased in 5-FU resistant cell lines than that in the corresponding parental cells, both in HCT116 cells and DLD1 cells. Knocking down TGFBI in HCT116 cells led to increased resistance to 5-FU treatment compared with control cells. GFP-TGFBI overexpression dramatically restored the sensitivity of resistant HCT116 cells to 5-FU treatment compared with GFP overexpression.

## Data Availability Statement

Publicly available datasets were analyzed in this study. This data can be found here: https://www.ncbi.nlm.nih.gov/geo/query/acc.cgi?acc=GSE3964 https://www.ncbi.nlm.nih.gov/geo/query/acc.cgi?acc=GSE19860.

## Ethics Statement

This study was approved by the Ethical Committee of Central South University (China).

## Author Contributions

YW and KF conceived the concept, instructed data analysis, and revised manuscript. YW and QW conducted most data analysis and preparation of figures and table and wrote manuscript draft. YY, YC, and SL helped with some analysis and interpretation of data. KF reviewed the manuscript with input from all authors. All authors contributed to the article and approved the submitted version.

## Funding

This work was supported in part by grants 31900561 from National Natural Science Foundation of China, Innovation-driven Projects 2020CX016 from Central South University, Huxiang Talent Aggregation Project 2018RS3027 and 2019RS1010 from Hunan Provincial Science and Technology Department (China). YW was supported by Xiangya Hospital Central South University Postdoctoral Foundation. The funders had no role in study design, data collection and analysis, decision to publish, or preparation of the manuscript.

## Conflict of Interest

The authors declare that the research was conducted in the absence of any commercial or financial relationships that could be construed as a potential conflict of interest.

## Supplementary Material

Supplementary Figure 1 The expression of TGFBI between responders and non-responders who receiving modified FOLFOX6 therapy in GSE19860 (**p*<0.05).

## Abbreviations

ABC transports, ATP-binding cassette (ABC) transports; BP, biological processes; CC, cellular compartments; CRC, colorectal cancer; DPD, dihydropyridine dehydrogenase; FDR, false discovery rate; GEO, gene expression omnibus; GO, Gene Ontology; KEGG, Kyoto Encyclopedia of Genes and Genomes; MF, molecular functions; OS, overall survival; PPI, protein-protein interaction; TGFB, transforming growth factor β; TOM, topological overlap matrix; TP, thymidine phosphorylase; TS, thymidylate synthase; WGCNA, weighted gene co-expression network analysis; 5-FU, 5-fluorouracil.
