# Supplementary figures and images for: Identification of Hub Genes Associated With Sensitivity of 5-Fluorouracil Based Chemotherapy for Colorectal Cancer by Integrated Bioinformatics Analysis
Source: Front Oncol. 2021 Apr 12;11:604315. doi: 10.3389/fonc.2021.604315 (PMC8071956; doi:10.3389/fonc.2021.604315)

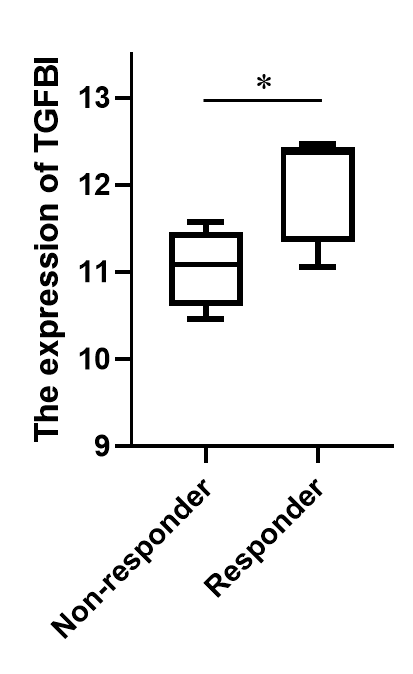

Supplement: Supplementary Figure 1 — The expression of TGFBI between responders and non-responders who receiving modified FOLFOX6 therapy in GSE19860 (*p<0.05). [file Image_1.tif]
